# Molecular Basis of Adenomatous Gastrointestinal Polyposis Syndromes: Role of Pathogenic and Benign Variants in Disease Onset

**DOI:** 10.3390/biomedicines14020426

**Published:** 2026-02-13

**Authors:** Francesca Cammarota, Valeria D’Agostino, Chiara Capasso, Francesca Duraturo, Valentina D’Angelo, Giovanni Battista Rossi, Paola Izzo, Rosario Vicidomini, Mimmo Turano, Marina De Rosa

**Affiliations:** 1 Department of Molecular Medicine and Medical Biotechnology, University of Naples Federico II, 80131 Naples, Italymarina.derosa@unina.it (M.D.R.); 2 Ceinge Biotecnologie Avanzate Franco Salvatore, 80131 Naples, Italy; 3 Division of Gastroenterology and Gastrointestinal Endoscopy, Istituto Nazionale Tumori-IRCCS “Fondazione G. Pascale”, 80131 Naples, Italy; 4 Section on Cellular Communication, Eunice Kennedy Shriver National Institute of Child Health and Human Development, National Institutes of Health, Bethesda, MD 20892, USA; 5 Department of Biology, University of Naples Federico II, 80126 Naples, Italy

**Keywords:** hereditary colorectal cancer, adenomatous gastrointestinal polyposis syndromes, genotype/phenotype correlation, next-generation sequencing, disease predisposing variants

## Abstract

**Background:** Colorectal cancer (CRC) is the third most diagnosed type of cancer and the second leading cause of cancer-related death. However, the increase in CRC incidence observed over the last 50 years has been accompanied by an overall reduction in mortality thanks to improved diagnostic strategies, patient follow-up, and more targeted therapies. Gastrointestinal adenomatous polyposis syndromes are a group of hereditary syndromes that predispose individuals to gastrointestinal tumors. These syndromes, characterized by the onset of gastrointestinal adenomas, are genetically heterogeneous. **Methods**: We analyzed 60 subjects with clinical suspicion or diagnosis of polyposis using next-generation sequencing (NGS). An additional 20 healthy individuals, all negative for pathogenic variants, were included in the study as a control population. We also performed bioinformatic analyses to investigate the hypothesis that benign variants could still be partially destructive, even though they cannot, by themselves, be responsible for the onset of disease. **Results:** Germline pathogenic variants were identified in 55% (33/60) of affected patients (MUT^+^), while variants of uncertain significance (VUS) were identified in 18.3% of affected patients (11/60). No variants were detected in the remaining 26.7% (16/60) of patients (MUT^−^). A genotype-phenotype correlation emerged from this study: MUT^+^ patients exhibited a significantly earlier age of onset and a higher number of polyps compared to VUS or MUT^−^ patients. Furthermore, Mendelian inheritance was significatively more frequent in MUT^+^ and VUS patients than in MUT^−^ individuals. Finally, the investigation of benign variants identified an SNP (single nucleotide polymorphism) of the APC gene promoter and a cluster of variants in POLD1, in which bioinformatic analysis predicted altered gene expression. **Conclusions:** These results suggest that, although MUT^−^ patients may develop multiple gastrointestinal adenomatous polyps, they are likely to have a familial predisposition rather than a Mendelian disorder. Furthermore, we propose that certain benign variants may be partially deleterious, potentially contributing to disease onset and/or act as phenotypic modifiers, likely through additive effects.

## 1. Introduction

Familial colorectal polyposis syndromes are a group of very rare hereditary disorders that are both phenotypically and genotypically heterogeneous and predispose individuals to colorectal tumor development [1,2,3,4]. Based on the histological characteristics of the polyps, colorectal polyposis syndromes are classified into adenomatous and hamartomatous types.

Adenomatous polyposis syndromes include familial adenomatous polyposis (FAP), attenuated FAP (AFAP), MUTYH-associated polyposis (MAP), and polymerase proofreading-associated polyposis (PPAP) [5].

Hamartomatous polyposis syndromes mainly include Peutz–Jeghers syndrome (PJS), juvenile polyposis syndrome (JPS) [6,7], and PTEN hamartoma tumor syndrome (PHTS) [6,8,9,10].

MAP is inherited in an autosomal recessive manner, whereas all other syndromes are reported to follow an autosomal dominant inheritance pattern [11,12].

Although hereditary polyposis syndromes are classically described as monogenic disorders, nearly fully penetrant and genotype–phenotype correlations are reported, and inter- and intra-familial phenotypic variability is also described. This heterogeneity may result from modifier alleles, somatic mutations, mosaicism, or other genetic and environmental factors and may complicate both diagnosis and clinical management [5].

The genes most commonly associated with adenomatous polyposis syndromes include *APC*, *MUTYH*, *NTHL1*, *POLE*, *POLD1*, and *AXIN2* [2,13,14]. Conversely, the genes mainly implicated in the onset of hamartomatous polyposis syndromes are *STK11*, *PTEN*, *BMPR1A*, *SDHB*, *SDHD*, *SMAD4*, *AKT1*, *ENG*, and *PIK3CA* [2,3,15,16,17].

FAP exhibits a broad phenotypic spectrum. The classic form is characterized by the development of hundreds to thousands of polyps, typically beginning around the age of 20. If untreated, these polyps inevitably progress to colorectal cancer, necessitating prophylactic colectomy. The attenuated form of FAP (AFAP) is marked by a later onset (around age 40) and a reduced polyp burden, usually fewer than 100 polyps. FAP patients often develop neoplasms in the upper gastrointestinal tract, such as gastric, fundic, duodenal, and ampullary adenomas, which represent the second leading cause of death after colorectal cancer (CRC). Other extraintestinal manifestations of FAP include osteomas, tooth anomalies, congenital hypertrophy of the retinal pigment epithelium (CHRPE), desmoid tumors, and extraintestinal tumors, such as thyroid, liver, bile duct, and central nervous system cancers [18,19,20].

MAP is characterized by the development of approximately 10–500 colorectal adenomas, with a lifetime risk of CRC between 43 and 48 years; the risk approaches 100% beyond the age of 48 [21]. On the other hand, monoallelic variants of MUTYH are associated with a moderate increase in CRC risk (1.5–2-fold), especially among individuals with a first-degree relative affected by CRC [21]. Individuals with MAP also face an elevated risk of duodenal cancer and non-melanoma skin cancer, as well as ovarian, bladder, and possibly endometrial cancers [22].

Individuals with PPAP may present with autosomal dominant inheritance, classical, or attenuated polyposis, CRC, and other somatic hypermutation-related tumors, even in the presence of a functioning DNA mismatch repair (MMR) system [14], including colorectal, endometrial, ovarian, breast, brain, and upper GI tumors.

The spectrum of adenomatous polyposis syndromes has recently been expanded to include two rare autosomal recessive conditions caused by biallelic mutations in NTHL1, a DNA glycosylase involved in base excision repair, and in MSH3, a gene involved in the MMR pathway [14,23]. Individuals carrying biallelic NTHL1 pathogenic variants frequently develop multiple independent tumors, highlighting the need for intensive, lifelong, and multi-system surveillance [22].

CRC screening has led to marked reductions in both cancer incidence and mortality over the past two decades [24]. In addition, family history, tumor histology, and molecular characterization are crucial for identifying individuals predisposed to CRC and to implement appropriate surveillance and treatments. Since differential clinical diagnosis can be difficult because overlapping features exist between polyposis syndromes, molecular diagnosis is pivotal for accurate classification and appropriate clinical management [20,25,26].

The primary aim of this study was to elucidate the molecular basis underlying the onset of familial adenomatous gastrointestinal polyposis syndromes and to identify the genes and mechanisms involved in their pathogenesis.

Achieving these goals will not only improve diagnostic accuracy but facilitate the identification of novel therapeutic targets for more effective diseases management.

We also sought to explore genotype–phenotype correlations by defining the clinical features of patients carrying or not carrying pathogenic variants or VUS (variants of uncertain significance), evaluating age at onset, number of colorectal polyps, and inheritance pattern (presence/absence of Mendelian autosomal inheritance). To this end, statistical analyses were performed on the studied population cohort, which was divided into the following three groups:

- Patients carrying a pathogenic/likely pathogenic germline variants (MUT+);
- Patients carrying a VUS germline variant (VUS);
- Patients without any germline variant, neither pathogenic nor VUS (MUT-).

As a secondary aim, we explored the potential contribution of benign variants, hypothesizing that such variants could lead to gene alteration through an additive effect, thereby contributing to disease onset. Previous studies, particularly genome-wide association studies, have shown that common low-penetrance variants, often classified as benign, can cumulatively modulate CRC risk [27].

## 2. Materials and Methods

### 2.1. Patients and Samples

Sixty subjects with clinical suspicion/diagnosis of adenomatous polyposis or subjects carrying pathogenic variants in genes associated with adenomatous polyposis syndromes without clinical evidence of polyposis but showing personal and familial cancer aggregation were enrolled in this study. Individuals heterozygous for monoallelic pathogenic variants in genes associated with hereditary recessive polyposis were included to explore incomplete or low-penetrance phenotypes and to reduce selection bias toward classical polyposis presentations. All probands included in this study were referred for molecular screening after a careful investigation of clinical history by a specialized clinician and genetic counseling. Inclusion criteria were the presence of multiple gastrointestinal adenomas and/or other neoplasm associated with the disease and/or positive family history for the disease. Patients arrived at the diagnostic laboratory of hereditary colorectal tumors (U.O.C. Clinical Molecular Biology) of the Federico II/CEINGE, University Hospital of Naples, between 2017 and 2023, for molecular diagnosis. Three samples of peripheral blood were obtained from all patients. DNA extraction from peripheral blood lymphocytes was carried out on two of the three blood test tubes drawn from each patient in order to obtain two different DNA aliquots, as previously described [15].

A population of 20 healthy subjects was also included into the study. This control population was recruited from unaffected members of at-risk families with previously negative results for the presence of the specific pathogenetic variant. All control subjects were matched for age (all adults) and were processed using the same workflow applied to individuals with suspected or confirmed adenomatous polyposis, including the use of the same sequencing panel and the same data analysis pipeline to minimize bias.

### 2.2. Molecular Screening of Gastrointestinal Polyposis Syndrome

**Figure 1:**
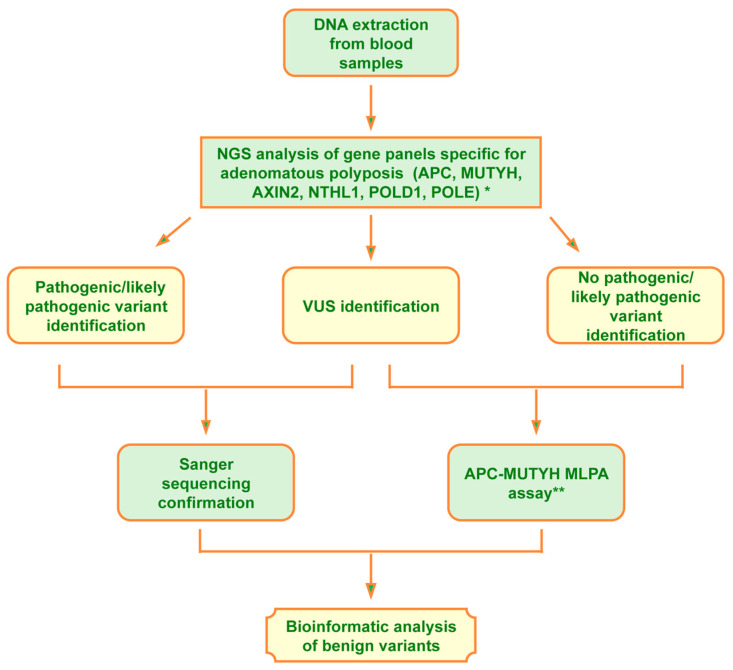
Workflow of adenomatous gastrointestinal polyposis molecular screening. *: Patients n° 13 and 44 were also analyzed for genes involved in hamartomatous polyposis because differential diagnosis between syndromes could not be performed. **: Only some of the patients who were offered the Multiplex Ligation-dependent Probe Amplification (MLPA) test agreed to undergo it.

To perform the molecular analysis of familial gastrointestinal polyposis, we set up the workflow reported in Figure 1.

The DNA extracted from the proband’s peripheral blood was first analyzed, using the next-generation sequencing (NGS) technique, for genes involved in adenomatous polyposis and, when pertinent (patients n° 13 and 44), also for genes involved in hamartomatous polyposis. Two gene panels, one specific for adenomatous polyposis syndromes, including 6 genes (*APC* (NM_000038.6), *AXIN2* (NM_004655.3), *MUTYH* (NM_001048174.2), *NTHL1* (NM_002528.7), *POLD1* (NM_002691.4), and *POLE* (NM_006231.4)) and another specific for hamartomatous polyposis, including 10 genes (*AKT1* (NM_001382430.1), *BMPR1A* (NM_004329.3), *CDH1* (NM_004360.5), *ENG* (NM_001114753.3), *PIK3CA* (NM_006218.4), *PTEN* (NM_000314.8), *SDHB* (NM_003000.3), *SDHD* (NM_003002.4), *SMAD4* (NM_005359.6), and *STK11*/*LKB1*(NM_000455.5)), as previously described [15], were analyzed via the next-generation sequencing technique using the AmpliSeq Library PLUS for Illumina Kit (catalog ID: 20019101, Illumina, San Diego, CA, USA) according to the manufacturer’s instructions. The pooled and barcoded libraries were subsequently sequenced using the MiSeq Sequencing System (Illumina, San Diego, CA, USA). Variant calling and analysis were performed using Base Space Sequence HUB/variant interpreter Software v7.41.0 (basespace.illumina.com, San Diego, CA, USA). The raw FASTQ files generated and/or analyzed during the current study are available on Mendeley data (De Rosa, Marina (2025), “Molecular screening of adenomatous gastrointestinal polyposis syndrome”, Mendeley Data, V1, doi: 10.17632/2wxnmhwkhm.1)

The interpretation of the identified variants was performed in accordance with ACMG guidelines [28] using Varsome free software v13.12.2 (varsome.com, Saphetor SA, Lausanne, Switzerland) ([29] and Franklin by Genoox free software v90.1 (franklin.genoox.com, Tel Aviv District, Israel). Reference databases for hereditary colorectal tumors, such as the InSight-group (www.insight-group.org) and ClinVar (www.ncbi.nlm.nih.gov/clinvar, accessed on 28 March 2025) databases, were also examined.

Pathogenic variants and/or variants of unknown pathogenic significance, identified via NGS, were confirmed through polymerase chain reaction (PCR) and Sanger sequencing performed on a second, independently extracted DNA sample, using the primer pairs previously reported for the APC, MUTYH, and STK11 genes [15,30], and primers reported in Table S1 for the other analyzed genes. The use of a second DNA aliquot is recommended in order to minimize the risk of technical artifacts, sample handling errors, or sample swaps. Finally, for the copy number variation (CNV) investigation, the Multiplex Ligation-dependent Probe Amplification (MLPA) assay was offered to subjects in which no pathogenic/likely pathogenic variants were identified, also given the high frequency of large deletions described in the APC gene.

### 2.3. Statistical Analysis

To investigate genotype–phenotype correlations, patients were classified into three groups based on the molecular findings: carriers of one or more pathogenic variants (MUT^+^), carriers of variants of uncertain significance (VUS), and individuals without any identified variants (MUT^−^). Three phenotypic variables were analyzed across mutation groups: age at disease onset (continuous), polyp burden (ordinal), and inheritance pattern, assessed either as a binary Mendelian versus non-Mendelian classification or further subdivided into inheritance categories (e.g., DOMINANT or RECESSIVE). Inheritance patterns were determined based on pedigree analysis and clinical family history. Age at disease onset was treated as a continuous variable. Normality within groups was assessed using the Shapiro–Wilk test, and homogeneity of variances was evaluated using Levene’s test. Group comparisons were performed using one-way analysis of variance (ANOVA), followed by Holm-adjusted pairwise t-tests when appropriate. Because the reported number of gastrointestinal polyps included approximate values (e.g., “<10” and “>100”) and non-numeric clinical annotations, polyp burden was categorized into clinically meaningful ordered intervals (0, <10, 10–20, 20–50, 50–100, >100, and >1000) and analyzed as an ordinal variable. Group differences in polyp burden were therefore evaluated using the Kruskal–Wallis rank-sum test, followed by Dunn’s post hoc test with Holm correction for multiple comparisons. Categorical variables were analyzed using Pearson’s Chi-squared test applied to contingency tables. When overall significance was detected, post hoc pairwise comparisons of proportions were conducted with Holm-adjusted *p*-values. Heatmaps were used to visualize both adjusted *p*-values and group-wise count distributions. All statistical tests were two-sided, and a *p*-value < 0.05 was considered statistically significant. Correction for multiple testing was consistently applied using the Holm method across all pairwise comparisons.

In addition to *p*-values, effect size measures were reported to support clinical interpretability. For one-way ANOVA, eta-squared (η^2^) was calculated to quantify the proportion of variance in age at onset explained by mutation group. For Kruskal–Wallis analyses of ordinal polyp burden, epsilon-squared (ε^2^) was computed as a non-parametric measure of effect size (ε^2^ = (H − k + 1)/(n − k), where H is the Kruskal–Wallis statistic, k is the number of groups, and n is the sample size). For Chi-squared tests assessing associations between mutation group and inheritance pattern, Cramér’s V was estimated. Effect sizes were interpreted according to conventional thresholds (small, medium, and large).

All statistical analyses and visualizations were performed using R (version 4.4.2), with functions from the base stats package and the car, FSA, effectsize, ggplot2, ggstatsplot, dplyr, tidyr, and tibble packages.

### 2.4. Bioinformatic Analysis

To further investigate if the benign/likely benign variants identified could play any role in disease onset, a heatmap was generated using Microsoft Excel software that plotted each variant identified during the screening of the adenomatous gene panel, for each of the 80 subjects analyzed for the purpose of this study, reporting the presence of the variant in red and its absence in blue.

A second variant map was obtained by subtracting all variants identified in the healthy population from variants identified in affected subjects analyzed into the study. Afterward, to investigate a possible deleterious effect of these benign/likely benign variants, they were analyzed with the following software: Human Splicing Finder and UMD-Predictor Pro from Genomics https://genomnis.com (accessed on 28 March 2025) and HaploReg v4.2 (https://pubs.broadinstitute.org/mammals/haploreg/haploreg.php, accessed on 28 March 2025), using default settings. Analyses from these three software were performed separately and the results were successively combined to obtain a consensus framework. This integrative approach supported the selection of candidate variants for future functional validation.

Human Splicing Finder (HSF) is a bioinformatics tool designed to analyze DNA or RNA sequences to predict the impact of mutations on the pre-mRNA splicing process.

It provides a predictive score to estimate the probability that a variant alters physiological splicing. The significance thresholds for each change are the following:

Splice site score → change ≥10% → possible functional alteration

Splice site creation → score > 65–70 → potentially active

ESE/ESS ratio → alteration ≥2 → possible effect on splicing

ESE/ESS ratio → alteration ≥4 → likely relevant effect

UMD-Predictor Pro software evaluates the pathogenicity of a DNA variant using a combinatorial algorithm that integrates several criteria.

The algorithm calculates an overall normalized score on a scale from 0 to 100. Based on this score, variants are classified as follows:

- Polymorphism (likely benign) → Score < 50;
- Likely polymorphism →       Score 50–64;
- Likely pathogenic mutation →   Score 65–74;
- Pathogenic mutation →      Score > 74 [31].

HaploReg v4.2 is a bioinformatics software designed for the functional analysis of genetic variants, mainly SNPs (single nucleotide polymorphisms).

The main functionalities are to provide information on histone modifications, chromatin state, transcription factor binding regions (TFBS), and DNase hypersensitivity, integrating data from projects such as ENCODE and Roadmap Epigenomics. This software also extends indexed SNPs to their SNPs in linkage disequilibrium (LD), using data from the 1000 Genomes Project for different populations, identifies potential regulatory effects of SNPs on target genes, such as expression quantitative trait loci (eQTLs), and assesses how a variant may alter transcription factor binding motifs, using motif databases such as TRANSFAC or JASPAR [32].

Together, these tools offer a comprehensive assessment of possible splicing, functional, and regulatory consequences, allowing a more complete interpretation of each variant’s potential impact.

Additionally, STRING analysis was performed to analyze the interactions between the following genes involved in the onset of gastrointestinal polyposis: *APC*, *MUTYH*, *AKT1*, *AXIN2*, *STK11*, *POLD1*, *POLE*, and *NTHL*. A STRING analysis is a bioinformatic approach that uses the STRING database to explore protein–protein interaction (PPI) networks. By inputting a list of genes or proteins, STRING identifies known and predicted interactions and visualizes them as a network [33,34].

## 3. Results

### 3.1. Main Findings

We established a molecular screening workflow for adenomatous gastrointestinal polyposis based on NGS analysis of a targeted gene panel, including *APC*, *MUTYH*, *POLE*, *NTHL1*, *AXIN2*, and *POLD1*. The selection of genes included in the multigene panel for adenomatous polyposis was based on the recommendations of the NCCN (National Comprehensive Cancer Network) [35], the ESMO (European Society of Medical Oncology) [36], the JSCCR (Japanese society for cancer of the colon and rectum) [37], and the ACMG (American College of Medical Genetics and Genomics) [38].

CNV analysis for *APC* and *MUTYH* genes was offered to patients without point pathogenic variants using the MLPA method.

When a differential diagnosis between adenomatous and hamartomatous polyposis could not be clearly established, patients were additionally analyzed using a specific panel targeting hamartomatous syndromes, including *PTEN*, *STK11*, *SDHB*, *SDHD*, *BMPR1A*, *CDH1*, *AKT1*, *SMAD4*, *PI3KCA*, and *ENG* genes. As suggested [39,40], probands who developed fewer than 10 adenomatous polyps but exhibited clinical features consistent with Lynch or Lynch-like syndromes were referred for MMR gene screening and excluded from this study.

As expected, the highest frequency of pathogenic variants was detected in *APC* and *MUTYH* genes, consistent with literature data [41,42]. By contrast, VUS were identified in a broader range of genes—*APC*, *MUTYH*, *NTHL1*, *AXIN2*, *POLE*, *POLD1* and *AKT1*—with a relatively uniform distribution. This may reflect the limited functional characterization of VUS to date, as well as the fact that most prior screening efforts in adenomatous polyposis patients have focused primarily on *APC* and *MUTYH*, without the inclusion of other relevant genes.

The interpretation of VUS in our study was still largely based on computational predictions and was not complemented by solid functional data; experimental laboratory assays will be necessary to establish their pathogenic relevance more reliably.

The statistical analysis of clinical and molecular data, reported in the Methods section, revealed genotype–phenotype correlations among the three groups (MUT^+^, VUS, and MUT^−^) into which the patient cohort was stratified.

Specifically, MUT^+^ patients exhibited an earlier age of onset and significantly higher polyp counts compared to both VUS and MUT^−^ patients.

VUS carriers also differed significantly from MUT- patients in terms of the frequency of Mendelian inheritance, defined on the basis of pedigree analysis and clinical family history, which was even higher than that observed in MUT^+^ patients (0.82 vs. 0.55), whereas MUT^−^ patients showed a markedly low frequency (0.063).

Although this study is limited by the relatively small sample size, mainly the limited control group, and restricted gene panel analyzed, two key findings emerged from the analysis of benign variants:

1. Patients n° 18, 25, 26, 31, and 42 harbored a cluster of variants in *POLD1*, some of which were reported to be in LD, and bioinformatic analysis suggested a strong potential to cause splicing alterations. At least one SNP in each LD group was predicted to affect splicing. Furthermore, these variants localized to active regulatory regions and may alter transcription factor binding, also raising the possibility of altered expression of neighboring genes. On the positive strand, downstream of *POLD1*, lies *MYBPC2*, *SPIB*, and *EMC10*; upstream on the negative strand, is *NAPSA*. *MYBPC2* encodes a myosin-binding protein involved in cardiomyopathies; *SPIB* encodes a lymphoid-specific transcription factor; *EMC10* promotes angiogenesis and endothelial proliferation; and *NAPSA* encodes a protease inhibitor proposed as a marker in lung and renal cancers.

2. Patients n° 18, 25, 26, 53, and 55 carried the rs78429131 (APC: c.-31T > G) variant, located in the *APC* gene promoter. Bioinformatic evidence supported its potential role in modulating *APC* gene expression.

**Figure 2:**
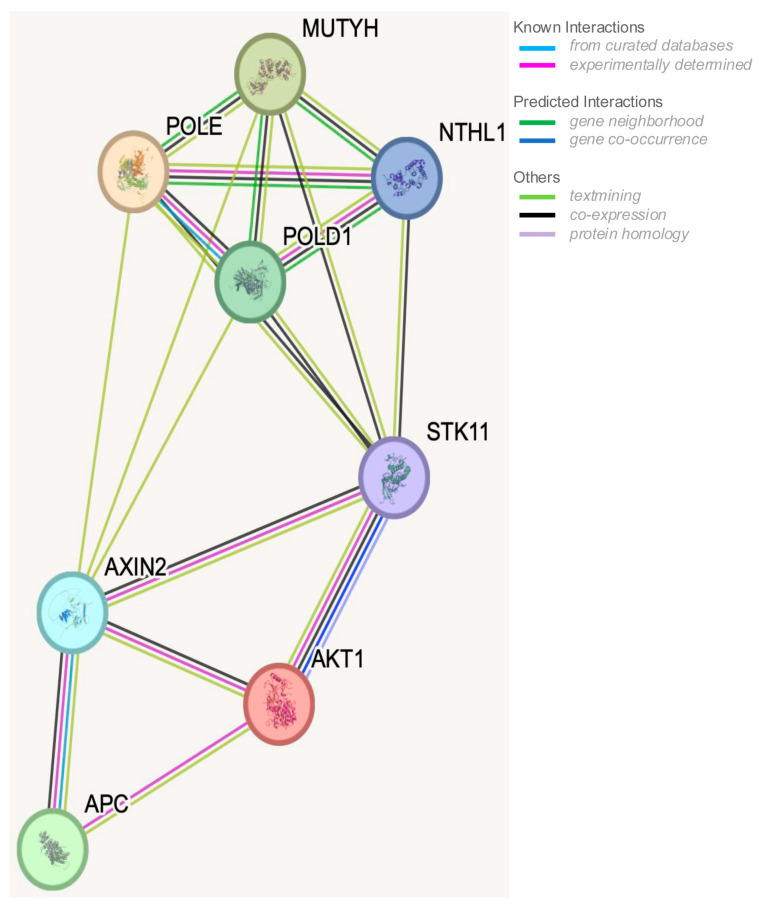
Crosstalk between genes involved in adenomatous gastrointestinal polyposis syndromes. Protein–protein interaction networks were analyzed using STRING (v12.0) [33,34], accessed on 7 May 2025. Edges represent specific and meaningful protein–protein associations. Proteins jointly contribute to a shared function; this does not necessarily mean they physically bind to each other. The STK11 gene was included in this analysis since it was functionally associated with several adenomatous gastrointestinal polyposis causative genes.

In this context, the possible crosstalk between gastrointestinal polyposis predisposing genes and molecular pathways, such as the BER system, Wnt signaling, and apoptosis, which contribute to shared cellular function, was investigated and is visually summarized in Figure 2, highlighting interactions among genes implicated in polyposis phenotypes.

Sixty patients were analyzed for adenomatous polyposis using genetic tests, as described in the Methods section.

We identified 33 patients (55%) carrying pathogenic variants in one of the analyzed genes, 11 patients (18.3%) harboring one or more variants of uncertain significance (VUS), and 16 patients (26.7%) were not informative, as they presented neither pathogenic/likely pathogenic nor VUS variants.

### 3.2. Genetic Findings (MUT^+^ Patients)

Among the 33 MUT^+^ patients, pathogenic variants were detected in the following genes: 21 in the *APC* gene, 1 in the *AXIN2* gene, 10 in the *MUTYH* gene (with 17 distinct variants), and 1 in the *NTHL1* gene, as reported in Table 1.

In total, the 40 identified pathogenic variants included:

- Eleven frameshift and indel variants (27.5%);
- Nine nonsense variants (22.5%);
- Four splicing variants (10%);
- Nine missense variants (22.5%);
- Four in-frame deletion variants (10%);
- Three large deletions (7.5%).

### 3.3. Genotype–Phenotype Correlations (MUT^+^ Patients)

Among the 21 patients carrying an *APC* mutation, 11 exhibited an autosomal dominant inheritance pattern, while the remaining 10 showed no evidence of Mendelian transmission. Two of these were confirmed as de novo cases (Table 2, patients n° 4 and 12), while for the other 8 cases, de novo origin could not be confirmed because their apparently healthy parents declined molecular screening (Table 2, patients n° 1, 2, 5, 7–9, 11, 15).

Of the 10 patients carrying mutations in the *MUTYH* gene, 5 exhibited a recessive pattern of inheritance, as expected, while the remaining 5 showed no clear evidence of Mendelian transmission. Three patients were found to carry a homozygous pathogenic variant in *MUTYH* (Table 2, patients n° 28–30), but only one of them had documented parental consanguinity (Table 2, patient n° 29).

The average age at disease onset in MUT^+^ patients was approximately 41 years, and the average number of polyps exceeded 100. Approximately 54% of these patients showed a Mendelian pattern of inheritance, as reported in Table 2.

A detailed molecular and clinical description is provided in Appendix A.

### 3.4. Genetic Findings (VUS Patients)

Among patients carrying only VUS, 3 carried variants in the *APC* gene, 1 in the *MUTYH* gene, 2 in the *NTHL1* gene, 2 in the *AXIN2* gene, 1 in the *POLD1* gene, 1 in the *POLE* gene, and 1 in the *AKT1* gene.

All VUS variants were missense, except for one splicing variant in the *AKT* gene (Table 3, patient n° 44).

### 3.5. Genotype-Phenotype Correlations (VUS Patients)

In this group of patients, the average age of onset was approximately 53 years; the average number of polyps was about 10, and approximately 82% of patients had a Mendelian type of inheritance, as reported in Table 3.

A detailed description of the clinical features of these patients, along with the criteria used for clinical classification of each variant, is provided in Appendix B.

### 3.6. Genetic Findings (MUT^−^ Patients)

Sixteen patients were found to be not informative for the presence of either pathogenic variants or VUS in the analyzed gene panel.

### 3.7. Genotype-Phenotype Correlations (MUT^−^ Patients)

Among the MUT^−^ patients, the average age at disease onset was approximately 53 years; the average number of polyps was around 10, and only approximately 6% of patients exhibited a Mendelian pattern of inheritance, as reported in Table 4.

All patients presented with more than 10 adenomatous polyps, except for patient n° 52, who developed only 4 polyps.

### 3.8. Statistical Analysis Results

**Figure 3:**
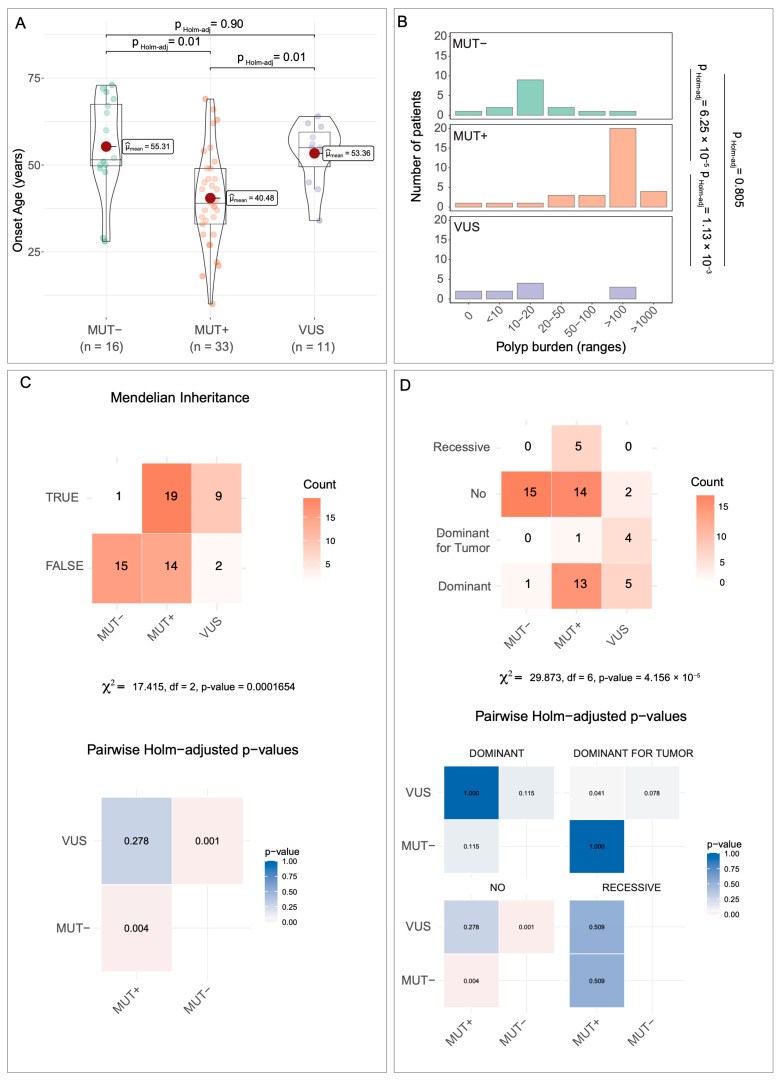
Statistical analysis of MUT^+^, VUS, and MUT^−^ patients. (**A**) Combined boxplot + violin plot of age at disease onset across mutation groups. One-way ANOVA (Welch correction) with Holm-adjusted pairwise comparisons showed a significant group effect (η^2^ = 0.23; 95% CI: 0.02–0.42). (**B**) Polyp burden is displayed as clinically defined ordinal ranges for mutation-negative (MUT^−^), mutation-positive (MUT^+^), and variants of uncertain significance (VUS) patients. Bars represent the number of patients per category. Group differences were assessed using the Kruskal–Wallis test (*p* = 7.76 × 10^−6^). Post hoc Dunn’s test with Holm correction showed a significantly higher polyp burden in MUT^+^ patients compared to MUT^−^ (*p* = 6.25 × 10^−5^) and VUS (*p* = 1.13 × 10^−3^) patients, whereas no significant difference was observed between MUT^−^ and VUS patients. The magnitude of this association was large (ε^2^ = 0.38), indicating that mutation class accounted for a substantial proportion of variability in polyp burden. (**C**) Heatmap of Holm-adjusted pairwise *p*-values for Mendelian inheritance (TRUE/FALSE) based on Pearson’s Chi-squared test, which revealed a significant association with mutation class (Cramér’s V = 0.50; 95% CI: 0.23–1.00). (**D**) Heatmap of post hoc standardized residuals and Holm-adjusted *p*-values for detailed Mendelian inheritance subclasses. Effect size measures are not reported for this multi-category contingency table due to sparse counts.

From the statistical analysis, performed as described in the Methods section, differences between the three groups of patients emerged. For age at onset (Figure 3A), MUT^+^ patients were significatively younger than both VUS and MUT^−^ patients (mean values of ~40, ~53, and ~55 years, respectively). The one-way ANOVA showed a significant group effect, and the associated effect size was large (η^2^ ≈ 0.23; 95% CI: 0.02–0.42), indicating that a substantial proportion of the variance in age at onset was explained by the mutation class.

For the number of gastrointestinal adenomatous polyps (Figure 3B), MUT^+^ patients developed a much higher polyp burden (>100 polyps) compared to VUS and MUT^−^ patients (>10 polyps). Polyp burden differed significantly among mutation groups (Kruskal–Wallis test, χ^2^(2) = 23.53, *p* = 7.76 × 10^−6^), with a large effect size (ε^2^ = 0.38), indicating that mutation class explained a substantial proportion of the variability in polyp burden.

Regarding Mendelian inheritance (Figure 3C), the three groups did not behave in a dependent manner (Chi-squared = 17.415, df = 2, *p* = 0.0001654). The effect size for this association was large (Cramér’s V ≈ 0.50; 95% CI ≈ 0.25–0.68), indicating that inheritance patterns strongly differed depending on mutation status. Specifically, only ~6% of MUT^−^ patients (1/16) showed a Mendelian inheritance pattern. By contrast, ~54.5% of MUT^+^ patients (18/33) displayed Mendelian transmission: 36.4% (12/33) dominant, 15% (5/33) recessive, and 3% (1/33) dominant for tumors. Among VUS patients, approximately 82% (9/11) exhibited dominant Mendelian inheritance; notably, 5 of the 9 patients had a gastrointestinal polyposis phenotype, and 4 had gastrointestinal tumors (Figure 3D) (Chi-squared = 29.873, df = 6, *p* = 4.156 × 10^−5^).

Effect size for categorical binary outcomes (Mendelian inheritance TRUE/FALSE) was quantified using Cramér’s V. For the contingency table with multiple inheritance subclasses, post hoc standardized residuals and Holm-adjusted *p*-values were used instead of global effect size, as recommended for sparse multi-category tables.

### 3.9. Bioinformatic Analysis Findings

We considered all benign or likely benign variants identified in the 80 subjects analyzed in this study and generated a heatmap illustrating the presence (red squares) or absence (blue squares) of these variants in each gene across subjects (Figure S1). This analysis was performed to understand whether benign or likely benign variants occurring in the same gene or gene network might have a cumulative impact on disease onset and/or phenotypic manifestations.

**Figure 4:**
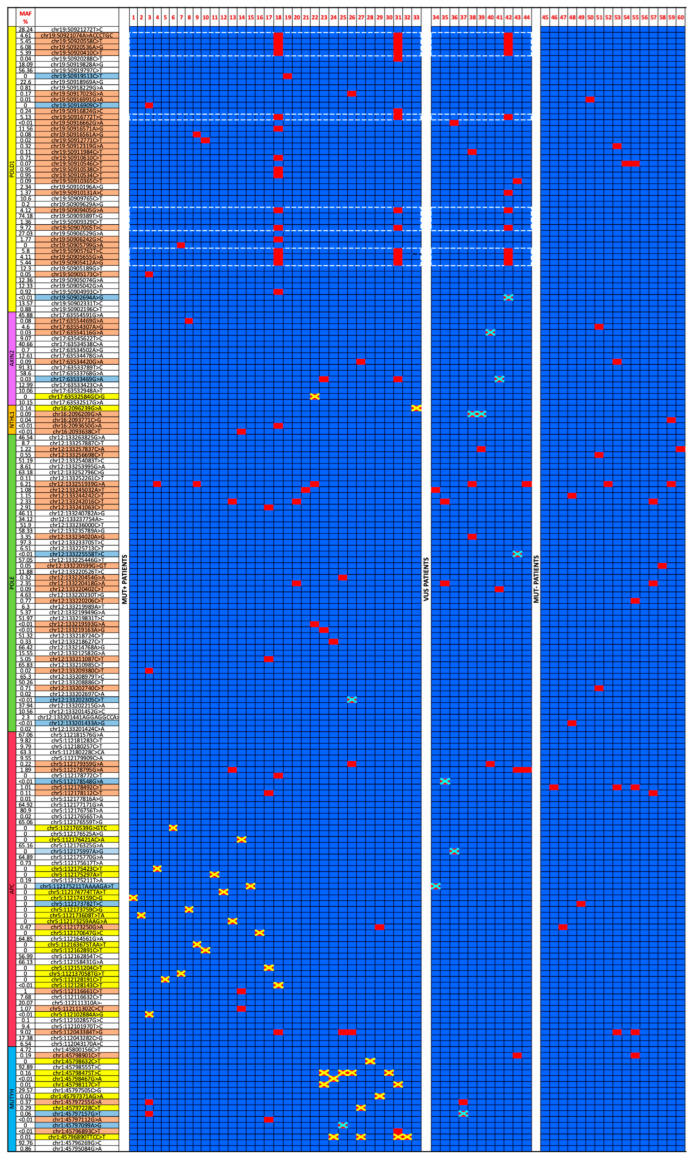
Variants heatmap. The heatmap shows variants identified in each patient that are not present in healthy control subjects. The presence or absence of the specific variant is reported with red and blue squares, respectively. Yellow crosses mark pathogenic variants, light blue crosses mark VUS. Variant positions are reported according to GRCh37 and GRCh38 position. Variants listed on the left side are highlighted as follows: orange for benign variants detected exclusively in affected subjects, white for benign variants detected exclusively in healthy subjects, yellow for pathogenic variants, and blue for VUS. The variants enclosed in the white dashed border refers to *POLD1* cluster variants.

Subsequently, variants observed in the unaffected control population were subtracted from those identified in patients, resulting in a refined mutational heatmap that displayed, for each patient, all variants not detected in healthy control individuals and their corresponding minor allele frequencies (MAFs) (Figure 4).

As shown in Figure 4, the majority of benign variants of affected subjects showed low MAF, with 58% of the variants displaying MAF <1%, 13% between 1% and 2%, and 29% between 2% and 11%. By contrast, healthy subjects exhibited a predominance of common variants, with 62% of subjects showing MAF >10%, 18% between 2% and 9%, while only 20% presented MAF < 1%.

Interestingly, no benign variants of the *NTHL1* gene were observed in healthy subjects and MAFs of benign variants identified in affected subjects was <0.1%.

This distribution supported a potential partially disruptive effect of benign variants in accordance with differences in allele frequency. Furthermore, the absence of benign variants in the *NTHL1* gene could indicate that the gene was functionally essential and therefore the variants assumed a pathogenic significance with high probability.

Each benign variant was analyzed using bioinformatic tools, as described in the Methods section.

Analysis of the refined heatmap revealed, in our opinion, three noteworthy observations, consisting of the identification of:

1. A cluster of benign variants in the *POLD1* gene in patients n° 18, 31, and 42.
2. The rs78429131 variant of the *APC* promoter region, named APC: c.-31T > G, in patients n° 18, 25, 26, 53, and 55.
3. Other benign variants identified in MUT^−^ patients were suggested to be partially disruptive.

The *POLD1* cluster variants (point 1) comprising the SNPs are listed in Table 5.

Results from HaploReg v4.2 analysis (a software examining functional consequences of SNPs on gene expression) of this cluster of variants identified two groups of SNPs in linkage disequilibrium (as reported in the Table 5), including the following: rs1726804, rs3212328, rs1143666, rs3218764, and rs1274607 (the first group), and rs3212330, rs2463239, rs2463238, and rs112856489 (the second group), all of which showed an r^2^ (coefficient of determination) ≥ 0.8.

In addition, analysis with the Human Splicing Finder (HSF) tool (a software examining the potential deleterious effect on SNPs on splicing mechanism) predicted significant alterations for SNPs rs3219384, rs1274607, and rs112856489.

- rs3219384 and rs1274607 (the latter being the only SNP in this cluster not in linkage disequilibrium with other SNPs) were predicted to significantly alter the enhancer/silencer ratio (ESE/ESS) by approximately +4-fold and −8-fold, respectively. These changes could result in exon skipping or intron retention events.
- rs112856489 was predicted to disrupt a wild-type acceptor splice site, with a decrease in splicing score of −33.09% (from 77.75 to 52.02), indicating a likely functional impact.

HaploReg v4.2 analysis also revealed that each of these polymorphisms mapped to DNase I hypersensitive sites (DHS)—regions of DNA devoid of nucleosomes and thus accessible to transcription factors. Furthermore, all SNPs overlapped with genomic regions marked by histone modifications characteristic of active enhancers or promoters, in a tissue-specific manner, as detailed in Table 6.

These findings suggested that the identified variants were located within putative regulatory regions, potentially modulating gene expression in specific tissues.

This hypothesis was supported by the observation that these SNPs can create or disrupt transcription factor binding motifs or alter the binding affinity of transcription factors for their target DNA sequences.

- On the negative DNA strand, the variants:
  ○ Create recognition sites for E2F, PU.1, SRF, Sin3A, TATA-box, CAC-binding protein, Egr-1, Ets, and SP1;
  ○ Disrupt sites for Sin3A, BCL, and ZBTB7A;
  ○ Increase affinity for SP1 and STAT binding motifs.
- On the positive DNA strand, the variants:
  ○ Create recognition sites for p300 and RXRA;
  ○ Increase affinity for motifs bound by GLI, NF-κB, NRSF, and CCNT2.

Furthermore, SNP rs1274607 mapped to a highly conserved genomic region. Several of the analyzed SNPs were located within specific DNA-protein binding sites, including:

- rs1726804 within a ZNF263 binding site.
- rs3212330, rs2463239, and rs2463238 within a POL2 binding site.
- rs3212330, also within a ZEB1 binding site.

The rs78429131 variant (APC: c.-31T > G) (point 2), located at position chr5-112043384 (GRCh37), was observed in patients n° 18, 25, 26, 53, and 55. It showed a population frequency of 0% according to the GnomAD database.

HaploReg v4.2 analysis suggested a possible deleterious effect on gene/protein expression of this SNP. This variant was indeed classified as an expression quantitative trait loci (eQTL) and was located within a Dnase I hypersensitive site, overlapping histone modifications associated with active promoter regions (TSSA; PROM_D1) and regulatory elements such as H3k4me1_Enh, H3K4me3_Pro, H3K27ac_Enh, and H3K9ac_Pro, all in a tissue-specific manner.

These chromatin marks are particularly enriched in gastrointestinal tissues, including:

- Colon mucosa and smooth muscle;
- Duodenal mucosa and smooth muscle;
- Esophagus;
- Rectal mucosa and smooth muscle;
- Sigmoid colon;
- Small intestine.

The variant also overlapped with binding sites for several transcription factors, including POL2, POL24H8, SIN3A, OCT2, POU2F2, and NFKB. Importantly, the presence of the rs78429131 polymorphism created a de novo binding site for the transcription factor HMX1, a transcription factor belonging to the H6 family of homeobox proteins, which often acts as a transcriptional repressor of genes involved in the developmental morphogenesis of the eye and specific nervous system structures.

Unfortunately, due to the lack of RNA and protein extracts from these patients, we were unable to perform additional molecular analyses to assess the functional consequences of these variants.

Finally, since MUT^−^ patients appeared to represent a sub-population with familial predisposition to the disease, rather than individuals affected by a Mendelian disorder, we conducted a bioinformatic analysis on benign variants identified in these patients that were absent in healthy controls (point 3). As a result of this analysis, we highlight the following notable findings:

- Patient n° 48 carried the *POLE* variant c.2174-8G > A, which was predicted using HSF (Human Splicing Finder) software to potentially alter splicing by activating a cryptic splice donor site, with a score variation of 18.56% (from 55.45 to 65.74).
- Patient n° 51 harbored two *POLE* variants classified as benign: c.6494G > A and c.330 + 66G > A. Both were predicted to cause splicing alterations by HSF.
  ○ The first variant would significantly alter the ESE/ESS motif ratio (−2);
  ○ The second would activate a cryptic splice site, with a score variation of 53.64% (from 51.96 to 79.83);
  ○ Moreover, *POLE* c.6494G > A was classified as probably pathogenic by the UMD predictor, with a score of 67.
- Patient n° 53 carried the *POLD1* variant c.1893-60G > A, which was predicted to cause activation of a cryptic splice acceptor site, with a score variation of 73.71% (from 37.81 to 65.68) according to HSF.
- Patient n° 55 carried the *MUTYH* variant c.304 + 56G > A, also found in patient n° 43, which was predicted to activate a cryptic splice acceptor site, with a score variation of 64.6% (from 43.14 to 71.01) by HSF.
  This patient also carried the rs78429131 variant (*APC*: c.-31T > G), as previously discussed.
- Patient n° 60 carried the *POLE* variant c.91G > T, also found in patient n° 39 (GnomAD frequency: 1.22%), and predicted by HSF to be potentially deleterious and to activate a cryptic donor site, with a score variation of 71.03% (from 38.21 to 65.35).

All of these variants were suggested to disrupt gene expression through a mechanism involving splicing alterations. Of these patients, only patient n° 53 showed evidence of Mendelian inheritance.

## 4. Discussion

In agreement with the results of molecular and statistical analyses, we propose that the VUS identified in this study may contribute to disease onset, likely resulting in a milder phenotype but still following a Mendelian inheritance pattern. Conversely, MUT^−^ patients exhibit phenotypic similarities to VUS carriers but have a much lower incidence of Mendelian inheritance.

Effect size estimates derived from our statistical analyses further reinforced these observations. The large effect sizes for age at onset (η^2^ ≈ 0.23) and polyp burden (ε^2^ ≈ 0.38) indicated that mutation class accounts for a substantial proportion of phenotypic variability. Likewise, the strong association between mutation status and Mendelian inheritance (Cramér’s V ≈ 0.50) demonstrated that inheritance patterns differ markedly across patient subgroups. Together, these measures complement traditional significance testing and support the interpretation that MUT^+^, VUS, and MUT^−^ individuals represent biologically distinct categories.

This supports the hypothesis that MUT- patients represent cases with familial predisposition or sporadic polyposis, rather than true Mendelian disease. It is likely that these patients do not carry any single variant sufficient to cause disease, but the disease phenotype is the result of additive effects of multiple partially disrupting variants.

Based on the results obtained from the bioinformatic analyses, we hypothesize a possible contribution of the cluster variant polymorphisms in the POLD1 gene and other benign variants in the *APC* gene (the rs78429131 variant; *APC*: c.-31T > G), but also in the *POLE* and *MUTYH* genes, to disease onset, progression, or phenotype, potentially through additive effects involving multiple molecular mechanisms. To confirm this hypothesis, further studies analyzing the mRNA and protein expression of these genes, as well as investigating the additive or synergistic effects of co-occurring benign or VUS variants through a systems biology approach, are needed.

The pathogenicity of monoallelic *MUTYH* and *NTHL1* variants remains controversial. Monoallelic germline *MUTYH* mutations have long been known to increase lifetime cancer risk [43,44,45]. However, risk estimates for monoallelic carriers remain variable across populations and study designs, suggesting the influence of additional genetic or environmental modifiers.

While earlier reports suggested that monoallelic *NTHL1* variants were not associated with increased tumor or polyposis risk [46,47], more recent evidence shows that tumors with biallelic and monoallelic *NTHL1* mutations share somatic mutational patterns [48,49], and monoallelic *NTHL1* variants may elevate lifetime cancer risk [46,50]. It is conceivable that, in some patients, pathogenic phenotypes arise from the additive effects of monoallelic variants in recessive genes such as *MUTYH* and *NTHL1*, combined with additional mildly deleterious variants in the same or other genes.

Finally, we hypothesize that the MUT^−^ patient group may represent cases in which disease onset results from a cumulative burden of multiple partially deleterious variants in crosstalking predisposing genes and interacting molecular pathways that contribute to shared cellular function.

In conclusion, our findings support distinct etiologies between MUT^+^ and VUS patients compared to MUT^−^ individuals. We propose that benign variants could represent mildly deleterious variants, that either singly or in combination, may act as disease modifiers, contributing to polyposis risk via additive effects across shared molecular pathways. Future studies with expanded gene panels and larger cohorts, including functional analysis of genomic variants, are needed to validate these hypotheses.

In light of these observations, this work presents an important innovative contribution and suggests that the interpretation of gene variants cannot be fully understood by the pathogenic/benign dichotomy. Our results support a model in which benign variants in specific genes, such as *POLD1* and APC, are not biologically irrelevant but can nevertheless have a disruptive impact on the gene or protein of variable entity. These variants, although not able to cause the disease phenotype by themselves, can determine quantitative modulations of gene expression or protein activity. The disease phenotype could result from the combined effects of multiple variants in the same gene or in genes of the same molecular pathway or crosstalking pathways. In this context, these variants could modulate the expression of the phenotype, such as disease penetrance or disease severity, expressed in terms of age of onset and number of polyps developed by patients. Our findings also highlight the need to consider the effect that variants have on the regulation of gene expression, and not just on protein function, as programmed in the main tools that define the classification of gene variants. Recognizing their potential roles as modifiers of certain variants could improve phenotype prediction and risk stratification.

### Future Perspective

The results obtained from this study open up interesting future perspectives for translational research. First of all, it will be necessary to confirm the etiological distinction of the disease between MUT^+^, MUT^−^, and VUS patients through multicenter studies. Simultaneously, it will be necessary to enlarge the gene panels screened in patients with polyposis or consider a whole-genome approach. This will potentially allow the identification of new genes responsible for the onset of disease, as well as the cumulative contribution of benign variants. In vitro and in vivo studies in cellular and animal models will be necessary for a functional analysis of VUS variants and those classified as benign variants. Finally, polygenic risk models should be considered to assess the additive or synergistic role of partially deleterious variants. A better understanding of the etiology of the disease, as well as the role that only partially destructive variants may play, could influence diagnostic interpretation, risk stratification, and personalized surveillance strategies in patients with polyposis.

## Figures and Tables

**Table 1:** Distribution of pathogenic variants identified between the main genes responsible for hereditary adenomatous gastrointestinal polyposis syndromes.

Gene (Disease)	Pathogenic Variants	MUT^+^ Patients (%)
APC (Adenomatous_FAP)	21	21 (32.8%)
AXIN2 (Adenomatous_FAP)	1	1 (1.6%)
MUTYH (Adenomatous_MAP)	17	10 (16.4%)
NTHL1 (Adenomatous_MAP)	1	1 (1.6%)
TOTAL NUMBER OF PATHOGENIC VARIANTS—% OF MUT+ PATIENTS	40	33 (52.5%)

**Table 2:** Molecular and clinical features in adenomatous polyposis patients carrier of pathogenic variants (MUT^+^). “*” refers to a stop codon.

P.	Onset Age (Years)	Polyps Number	Mendelian Inheritance	Pathogenic Variant	Other Clinical Manifestations
1	39	>100	NO	*APC*_c.2868 C > G; p.Tyr956*	
2	43	>1000	NO	*APC*_c.2320insA; p.Asp774Glufs*14	
3	63	>100	DOMINANT	*APC*_c.221-2 A > G	
4	10	>100	NO	*APC*_c.4132C > T; p.Gln1378*	de novo mutation
5	46	>100	NO	*APC*_c.694C > T; p.Arg232*	
6	49	>100	DOMINANT	*APC*_c.5249_5250dupTC; p.Gln1751Serfs*16	
7	30	>1000	NO	*APC*_c.814del; p.Ala272Glnfs*21	
8	46	>100	NO	*APC*_c.2468C > G; p.Ser823*	
9	30	>100	NO	*APC*_c.1601_1602delAA; p.Lys534Ilefs*2	
10	22	>100	DOMINANT	*APC*_c.1495C > T; p.Arg499*	
11	33	>100	NO	APC_c.4006A > T; p.Arg1336*	
12	37	>100	NO	APC_c.3486_3487delTA; p.Tyr1162_Ser1163delins*	de novo mutation
13	21	<10	DOMINANT	*APC*_c.1974_1975del; p.Asn659Glnfs*14 *STK11*_c.1211C > T; p.Ser404Phe (VUS-conflicting interpretation)	Personal and family history of mixed polyposis of the large intestine, associated with malformative stigmata (bladder malformations, sebaceous cysts, and skin spots).
14	55	20–50	DOMINANT	*APC*_c.5132delC; p.Pro1711Leufs*33	
15	18	50–100	NO	*APC*_ c.3927_3931del, p.(Glu1309Aspfs*4)	
16	38	>1000	DOMINANT	*APC*_c.1744-1G > C	
17	38	>100	DOMINANT	*APC*_ c.847 C > T; p.Arg283*	A paternal cousin developed colon cancer.
18	44	>100	DOMINANT	*APC*_c.646C > T; p.Arg216*	The proband’s 8-year-old son was affected by hepatoblastoma; the proband’s mother died at the age of 43 from rectal cancer.
19	35	>1000	DOMINANT	*APC*_Del_exons 1_18	
20	27	>100	DOMINANT	*APC*_Del_exons 9_10	Carrier of a reciprocal translocation, apparently balanced: 46, XX, t (3; 8) (p14; q12).
21	27	>100	DOMINANT	*APC*_g.(?_112707441)_(112707900_?)del (1B promoter)	
22	66	50–100	DOMINANT	*AXIN2*_c.1994delG; p.Gly665Alafs*24	
23	40	>100	NO	*MUTYH*_c.536A > G; p.Tyr179Cys *MUTYH*_c. 734G > A; p.Arg245His	
24	54	>100	RECESSIVE	*MUTYH*_c.544C > T; p.Arg182Cys *MUTYH*_c.1437_1439delGGA; p. Glu480del	
25	37	>100	RECESSIVE	*MUTYH*_c.536A > G; p.Tyr179Cys *MUTYH*_c.1316T > C; p.Leu439Pro (VUS)	
26	51	>100	RECESSIVE	*MUTYH*_c.536A > G; p.Tyr179Cys *POLE*_ c.6583G > A; p.Asp2195Asn (VUS)	
27	34	20–50	NO	*MUTYH*_c. 1187 G > A; p.Gly396Asp *MUTYH*_c. 1437 _1439 delGGA; p.Glu480del	
28	43	10–20	RECESSIVE	*MUTYH*_c.463-1 G > A (Homozygous)	Non-consanguineous parents.
29	49	>100	RECESSIVE	*MUTYH*_c.1147delC; p.Ala385Profs*23 (Homozygous)	Consanguineous parents.
30	35	>100	NO	*MUTYH*_c.536A > G; p.Tyr179Cys (Homozygous)	Non-consanguineous parents.
31	62	50–100	NO	*MUTYH*_c.734G > A; p.Arg245His *MUTYH*_c.1437_1439 del; p.Glu480del	
32	45	20–50	NO	*MUTYH*_c.1437_1439 del; p.Glu480del	Family history of pancreatic and lung cancer in the maternal line.
33	69	NO	DOMINANT FOR TUMOUR	*NTHL1*_c.244C > T; p.Gln82*	The proband developed gastric adenocarcinoma showing MSS tumor phenotype and a calf melanoma. The proband reported a positive family history of myeloma (mother), thyroid cancer, and breast cancer (sister).
	About 40.48 (10–69)	>100	about 54%		

**Table 3:** Molecular and clinical features in adenomatous polyposis patients carrier of variants of uncertain significance (VUS).

P.	Onset Age (Years)	Adenomatous Polyps Number	Mendelian Inheritance	Variant of Unknown Significance	Other Clinical Manifestations
34	64	<10	DOMINANT	*APC*_c.3920T > A; p.Ile1307Lys	Hyperthyroidism
35	62	10–20	NO	*APC*_c.7257G > A; p.Met2419Ile	NONE
36	43	>100	DOMINANT	*APC*_C.4706G > A; p.Asp1569Gly	NONE
37	58	>100	DOMINANT FOR TUMOR	*MUTYH*_c.1258C > A; p.Leu420Met	The proband’s mother developed gastrointestinal polyposis at the age of 80 years; the proband’s brother developed a rectal cancer at the age of 60 years.
38	55	10–20	NO	*NTHL1*_c.274C > T; p.Arg92Cys;	The proband underwent surgery for melanoma at the age of 53; he and his father presented congenital infrarenal aortic aneurysms.
39	55	NO	DOMINANT FOR TUMOR	*NTHL1*_c.274C > T; p.Arg92Cys;	The proband developed uterine cancer at the age of 55; she was affected by ulcerative colitis with inflammatory polyps, lipomas, obesity, and insulin-resistant diabetes. The proband’s sibling showed unclassified intestinal polyps, lipomas, and prostate calcifying lesions; her daughter developed a thyroid cancer at the age of 25.
40	61	10–20	DOMINANT	*AXIN2*_c.623C > T; 40p.Ala208Val	NO
41	34	NO	DOMINANT FOR TUMOR	*AXIN2*_c.1685C > T; p.Pro562Leu	The proband developed breast cancer at the age of 34, endometrial cancer and rectal cancer at the age of 48. She reported a positive family history of breast, rectum, colon, endometrial, and thyroid disease. Her father and a paternal uncle developed gastrointestinal polyposis.
42	45	>100	DOMINANT	*POLD1*_c.269A > G; p.Gly90Arg;	NONE
43	54	<10	DOMINANT	*POLE*_c.4106A > G; p.Asn1369Ser	NONE
44	56	10–20	DOMINANT FOR TUMOR	*AKT1* _c.1260 + 5G > A	NONE
	53, 36 (34–64)	>10	About 82%		

**Table 4:** Molecular and clinical features in adenomatous polyposis patients without pathogenic or VUS variants identified (MUT^−^).

P.	Onset Age (Years)	Polyps Number	Mendelian Inheritance	Other Phenotipycal Manifestations
45	48	50–100	NO	NO
46	71	20–50	NO	Colon adenocarcinoma; diabetes; lung cancer at the age of 65; and a family history of lung and breast cancer.
47	65	10–20	NO	Colon adenocarcinoma; thyroid nodules; her father developed pancreatic cancer at the age of 75; and a maternal aunt developed biliary tract cancer at the age of 57.
48	51	10–20	NO	The proband’s father developed stomach cancer at the age of about 70 years; and a paternal uncle developed melanoma at the age of about 80 years.
49	60	10–20	NO	NO
50	51	20–50	NO	NO
51	28	>100	NO	NO
52	69	<10	NO	Rectal adenocarcinoma
53	29	10–20	DOMINANT	NO
54	52	NO	NO	The proband developed a colon adenocarcinoma; the proband’s mother developed a colon adenocarcinoma at the age of 52 years and her grandmother at an older age; an aunt of the proband instead developed thyroid cancer at the age of 62; and another aunt developed 3 colon polyps.
55	73	10–20	NO	The proband’s daughter developed uterus polyps
56	49	10–20	NO	The proband’s maternal aunt developed colon cancer at the age of 85; and a sister developed two colon polyps and uterine cancer at the age of 45.
57	50	10–20	NO	The proband showed a serrated-hyperplastic polyposis; and a family history of breast cancer (proband’s sister and daughter).
58	72	10–20	NO	NO
59	67	<10	NO	The proband’s mother developed colon cancer at the age of 72.
60	50	10–20	NO	The proband’s two sisters developed gynecological malignancies at the age of 12 and 49 years, respectively.
	About 55.31	>10	about 6%	NO

**Table 5:** *POLD1* SNP (single nucleotide polymorphism) cluster identified in patients n° 18, 25, 26, 31, and 42. Each polymorphism is reported with its GRCh37 position, GRCh38 position, HGMD nomenclature, SNP ID, and gnomAD population frequency/MAF. The * and § symbols mark the two groups of SNPs in linkage disequilibrium.

SNP ID	GRCh37 Position	GRCh38 Position	HGMD Nomenclature	gnomAD Population Frequency/MAF
rs1726804 *	chr19-50905412	chr19-50402155	*POLD1*:c.589 + 31A > G	5.44%
rs3212328 *	chr19-50905655	chr19-50402398	*POLD1*:c.758 + 25G > A	4.11%
rs1143666 *	chr19-50905762	chr19-50402505	*POLD1*:c.810T > C; p.ALA270=	5.8%
rs3219384	chr19-50907005	chr19-50403748	*POLD1*:c.1242 + 151T > C	9.72%
rs3218764 *	chr19-50909405	chr19-50406148	*POLD1*:c.1243-34G > A	4.12%
rs1274607 *	chr19-50916772	chr19-50413515	*POLD1*:c.2244T > C; p.Ser748	5.13%
rs3212330 ^§^	chr19-50920410	chr19-50417153	*POLD1*:c.3121-19C > T	5.39%
rs2463239 ^§^	chr19-50920536	chr19-50417279	*POLD1*:c.3218 + 10A > G	6.08%
rs2463238 ^§^	chr19-50920558	chr19-50417301	*POLD1*:c.3218 + 32C > T	5.45%
rs112856489 ^§^	chr19-50921074	chr19-50417817	*POLD1*:c.3219-25_3219-19dup	4.61%

**Table 6:** Effect of variants on DNase hypersensitivity, histone modifications, chromatin state, and transcription factor binding regions (TFBS), evaluated using HaploReg software. The * and § symbols mark the two groups of SNPs in linkage disequilibrium.

SNP ID	SiPhy cons	DHS	Protein Bound	Promoter Histone Marks	Enhancer Histone Marks	Regulatory Motif Changed (+ Strand): Ref./Alt.	Regulatory Motif Changed (− Strand): Ref./Alt.
rs1726804 *	none	presence	ZNF263		HEK9ac	GLI: 4/12.1; NF-KB: 3.6/15.5;	
rs3212328 *	none	presence			H3K9ac		SP1: 3.6/12.1
rs1143666 *	none	presence			H3K27ac_Enh; H3K9ac;		E2F: 0.2/12.1
rs3219384	none	presence			H3K27ac_Enh; H3K9ac; H3K4me1_Enh		PU.1: 0.3/11.7; SRF: −0.7/10.8; TATA: 0.3/12.2
rs3218764 *	none	presence			H3K9ac; H3K27ac	RXRA: −3.9/7.7	BDP1: 2.7/13.5
rs1274607 *	highly	presence		H3K9ac_Pro	H3K27ac; H3K4me1_Enh	NRSF: 2.3/11.6	Sin3Ak-20:0.1/11.1
rs3212330 ^§^	none	presence	POL2; ZEB1	H3K9ac_Pro	H3K27a2c_Enh; H3K4me1_Enh; 12_EnhBiv; 17_EnhW		Sin3Ak-20: 10.6/−0.9
rs2463239 ^§^	none	presence	POL2	H3K9ac_Pro	H3K27a2c_Enh; H3K4me1_Enh; 12_EnhBiv; 17_EnhW	P300: 0/11.4	
rs2463238 ^§^	none	presence	POL2	H3K9ac_Pro	H3K27a2c_Enh; H3K4me1_Enh; 12_EnhBiv; 17_EnhW		BCL: 12.7/0.7; ZBTB7A: 14/2.1;
rs112856489 ^§^	none	presence		H3K9ac_Pro H3K4me3_Pro	H3K27a2c_Enh; H3K4me1_Enh;	CCNT2: 1.7/13.7;	CAC-binding-protein: −10.9/12.5; Egr-1: −5.8/6.1; Ets: 0.6/10.5; SP1: −0.8/11 STAT: 2.1/12.8;

## Data Availability

The datasets generated and/or analyzed during the current study are available on Mendeley data (De Rosa, Marina (2025), “Molecular screening of adenomatous gastrointestinal polyposis syndrome”, Mendeley Data, V1, doi: 10.17632/2wxnmhwkhm.1).

## Supplementary Materials

The following supporting information can be downloaded at: https://www.mdpi.com/article/10.3390/biomedicines14020426/s1, Table S1: Oligonucleotides designed for PCR (polymerase chain reaction) and Sanger sequences of POLE, NTHL1, and AXIN2; Figure S1: Total variants heatmap.

## Appendix A. Molecular and Clinical Description of Most Significant Case Report—MUT^+^ Patients

Patient n° 13 was a woman of 21 years at the age of diagnosis who was referred for molecular screening with a diagnostic suggestion of gastrointestinal polyposis. She developed gastric, duodenal, and intestinal adenomas. She also showed a congenital malformation of the bladder valve, sebaceous cysts, and skin spots. The proband’s parents both had dental cysts, but they had not developed polyps nor any neoplastic disease. The proband’s maternal grandfather and grandmother developed biliary tract carcinoma at the age of 77 years and pancreatic carcinoma at the age of 56 years, respectively. The proband’s maternal grandmother developed diabetes. The geneticist first asked for *PTEN* gene screening, given the presence of bladder malformations, skin spots, and diabetes, which however was negative for the presence of pathogenic variants. Only the analysis of the complete adenomatous and hamartomatous polyposis gene panels allowed the identification of two germline variants in the proband: a pathogenic variant in the *APC* gene and a VUS in the *STK11* gene. The *APC* pathogenic variant was identified the *APC*_c.1974_1975del variant. p.Asn659Glnfs*14 (as reported in Table 2), consists of a small deletion of 2 nucleotides at position 1974–1975, which creates the substitution of the amino acid Asn at position 659 with a Gln, and a frameshift, generating a premature stop codon 14 codons downstream. This genomic variant was not previously reported in databases, nor in the literature. In agreement with ACMG standards and guidelines for the interpretation of sequence variants [26], the Varsome tool (https://varsome.com), and Franklin by Genoox tool (https://franklin.genoox.com, accessed on 28 March 2025), it was classified as pathogenic. Indeed, there was 1 very strong, 2 moderate, and 1 supporting lines of evidence of pathogenicity: it was a null variant (frameshift) in the APC gene, for which loss-of-function is a known mechanism of disease (PVS1); it was located in a described mutational hotspot (PM1); and it was absent from the “1000 Genome” exome sequencing project (PM2).

Patient n° 13 was also a carrier of an *STK11*/*LKB1* variant, *STK11*_c.1211C > A; p.Ser404Phe, reported in the ClinVar database (https://www.ncbi.nlm.nih.gov/clinvar/, accessed on 28 March 2025) as a VUS, in agreement with ACMG standards and guidelines for the interpretation of sequence variants [27]. Indeed, it presents 2 supporting lines of evidence of benignity and 1 supporting line of evidence of pathogenicity: the variant is a missense variant in a gene (*STK11*/*LKB1*) for which primarily truncating variants are known to cause disease (BP1); multiple lines of computational evidence suggest no impact on the gene or gene product (BP4); and the variant is not found in gnomAD genomes, nor in gnomAD exomes (PM2).

Patient n° 22 showed a variant in the *AXIN2* gene, the c.1994delG; p.Gly665Alafs*24 variant, which is classified as pathogenic in the ClinVar database. This variant consists of a small deletion of 1 nucleotide at position 1994 of the *AXIN2* gene, which creates the substitution of the amino glycine at position 665 with an alanine, and a DNA frameshift, generating a premature stop codon 24 codons downstream. In agreement with ACMG standards and guidelines for the interpretation of sequence variants [27], it is classified as pathogenic. Indeed, there are 1 very strong, 1 moderate, and 1 supporting lines of evidence of pathogenicity: it is a null variant (frameshift) in the *AXIN2* gene, for which loss-of-function is a known mechanism of disease (PVS1); it has extremely low frequency in the gnomAD population database and “1000 Genome” exome sequencing project (PM2); and a reputable source reported the variant as pathogenic (PP5).

Patient n° 25 was a carrier of two *MUTYH* variants, c.536A > G; p.Tyr179Cys, classified as pathogenic in the ClinVar database, and c.1316T > C; p.Leu439Pro, classified as a VUS in the ClinVar database. This last variant is located in the coding exon 13 of the *MUTYH* gene and consists of a T to C substitution at nucleotide position 1316, which causes the substitution of tyrosine at codon 439 with a proline, an amino acid with similar properties. This amino acid position is well conserved in available vertebrate species and its alteration is predicted to be deleterious through in silico analysis. In agreement with ACMG standards and guidelines for the interpretation of sequence variants [27], it is classified as a VUS variant. Indeed, there are 1 moderate and 2 supporting lines of evidence of pathogenicity: it is detected in trans with a pathogenic variant in a recessive disorder (PM3); the variant is not found in gnomAD genomes, nor in gnomAD exomes (PM2); and multiple lines of computational evidence support a deleterious effect on the gene or gene product (PP3). This patient was also carrier of a benign variant in the *APC* promoter region (rs78429131, *APC*: c.-31T > G), which is predicted to have a deleterious impact on gene expression because it is located within a Dnase I hypersensitive site. Moreover, the rs78429131 SNP alters transcription factor binding, supporting a functional regulatory role. The proband showed a classical FAP phenotype, with more than 100 polyps at disease onset at the age of 38 years and a recessive manner of inheritance.

Patient n° 26 was a carrier of a pathogenic variant in the *MUTYH* gene, c.536A > G; p.Tyr179Cys, and a second variant in the *POLE* gene, c.6583G > A; p.Asp2195Asn, which is reported in the ClinVar database as a VUS variant. No other pathogenic variant (point mutations or CNVs) was identified in the genes analyzed with the adenomatous panel. The *POLE* sequence change, located in coding exon 47 of the *POLE* gene, replaces aspartic acid, which is an acidic and polar amino acid, with asparagine, which is a neutral and polar amino acid, at codon 2195 of the *POLE* protein, in a conserved amino acid position. In agreement with ACMG standards and guidelines for the interpretation of sequence variants [27], it is classified as a VUS variant. Indeed, it presents 1 supporting line of evidence of benignity and 1 supporting line of evidence of pathogenicity: out of 365 pathogenic variants in *POLE* gene, there are 20 pathogenic missense variants versus 345 pathogenic truncating variants (BP1); and the variant is not found in gnomAD genomes, nor in gnomAD exomes (PM2). This patient was also a carrier of the same benign variant in the *APC* promoter region (rs78429131, APC: c.-31T > G) as patient n° 25. The proband showed a typical MAP phenotype, with more than 100 polyps at disease onset at about 50 years of age and a recessive manner of inheritance.

Patient n° 32 was a carrier of a single heterozygous *MUTYH* pathogenic variant, without other pathogenic variants (point mutations or CNVs) identified in any of the genes analyzed with the adenomatous panel. He developed approximately 50 adenomatous polyps at the age of about 45 years and did not show familial inheritance for gastrointestinal polyposis but a family history of pancreatic and lung cancer in the maternal line.

Patient n° 33 was a carrier of a single heterozygous *NTHL1* pathogenic variant, without other pathogenic variants (point mutations or CNVs) identified in any of the genes analyzed with the adenomatous panel. He was a man of about 69 years at the age of diagnosis and did not develop adenomatous polyps nor did he show familial inheritance for gastrointestinal polyposis, while he developed gastric adenocarcinoma characterized by an MSS tumor phenotype and a calf melanoma. The proband reported a positive family history of myeloma (mother), thyroid cancer, and breast cancer (sister).

## Appendix B. Molecular and Clinical Description of Most Significant Case Report—VUS Patients

Patient n° 34 was a carrier of the *APC*_c.3920T > A; p.Ile1307Lys missense variant. The isoleucine residue is weakly conserved and there is a moderate physicochemical difference between isoleucine and lysine. However, the variant, already described in the international scientific literature, introduces at the DNA level a small oligo-adenine (A8) hypermutable region, which causes an increase in the frequency of somatic variants and consequently an increase in colorectal cancer risk [51,52]. Several studies report that this variant represents a risk allele for the onset of colorectal cancers in the Ashkenazi Jewish population [53]; however, there is not enough evidence on its pathogenic significance in the population not descending from Ashkenazi Jews. This variant is reported in the ClinVar database and classified as VUS/conflicting interpretation. However, in agreement with ACMG standards and guidelines for the interpretation of sequence variants [27], it is classified as a likely benign variant because it presents 2 strong and 1 supporting lines of evidence of benignity and 1 supporting line of evidence of pathogenicity. Indeed, the GnomAD exomes allele frequency (0.00124) is greater than the threshold derived from the 11 462 clinically reported variants in gene *APC* (0.000133), (BS1); it is observed in healthy adults (BS2); multiple lines of computational evidence suggest no impact on the gene or gene product (BP4); and a reputable source recently reported the variant as pathogenic, although the evidence is not available for the laboratory to perform an independent evaluation (PP5).

The patient carrier of this VUS was a woman of 64 years at the time of diagnosis who did not report Ashkenazi Jewish and Finnish origins and developed colon carcinoma and few adenomas. The woman, who reported a dominant family history of colon cancer, was also affected by hyperthyroidism.

Patient n° 35 was a carrier of the *APC*_c.7257G > A; p.Met2419Ile variant, which consists of a G to A transition, causing the substitution of the neutral and non-polar amino acid methionine with isoleucine, which is also a neutral and non-polar amino acid, at codon 2419 of the APC protein. It is reported in the ClinVar database as a VUS, in agreement with ACMG standards and guidelines for the interpretation of sequence variants [27]. Indeed, there is 1 supporting line of evidence of benignity and 1 supporting line of evidence of pathogenicity: the variant is not found in gnomAD genomes (PM2) and it is a missense variant in a gene for which primarily truncating variants are known to cause disease (about 79.5% non-VUS missense variants in gene *APC* are benign) (BP1). The proband was a man of about 62 years at the age of diagnosis. He developed more than 10 adenomatous polyps and did not show other clinical manifestations of FAP syndrome nor a family history of gastrointestinal polyposis or colorectal cancer.

Patient n° 36 was a carrier of the *APC*_c.4706G > A; p.Asp1569Gly variant, which consists of a G to A transition, causing the substitution of the polar amino acid asparagine with isoleucine, which is a neutral and non-polar amino acid, at codon 1569 of the APC protein. It is not reported in the ClinVar database, while it is classified, in agreement with ACMG standards and guidelines for the interpretation of sequence variants [27], as a VUS. Indeed, there is 1 supporting line of evidence of benignity and 1 supporting line of evidence of pathogenicity: the variant is not found in gnomAD genomes (PM2) and it is a missense variant in a gene for which primarily truncating variants are known to cause disease (about 79.5% non-VUS missense variants in gene *APC* are benign) (BP1).

The proband was a woman of 43 years at the age of diagnosis who developed more than 100 gastrointestinal adenomas and showed a positive family history of FAP and CRC from maternal lineage.

Patient n° 37 was carrier of only one heterozygous variant in the *MUTYH* gene, *MUTYH*_c.1258C > A p.(Leu420Met), which consists of a C to A transversion, causing the substitution of the amino acid leucine at position 420 with a methionine. This variant is reported in the ClinVar database and classified as a VUS, in agreement with ACMG standards and guidelines for the interpretation of sequence variants [27]. Indeed, there are 1 moderate and 3 supporting lines of evidence of pathogenicity and 1 strong line of evidence of benignity: this variant is located in a mutational hotspot and/or critical and well-established functional domain without a benign variation mutational hotspot, specifically, it is located in the A domain of DNA_Glycosylase_C (R354-483Y aa) of the Adenine DNA glycosylation protein, whose non-VUS coding variants have 50% pathogenicity (PM1); it is a missense variant in a gene that has a low rate of benign missense variation and in which missense variants are a common mechanism of disease (68,3% of non-VUS missense variants in *MUTYH* gene are pathogenic variant, more than the threshold of 51.0%) (PP2); multiple lines of computational evidence support a deleterious effect on the gene or gene product (7 pathogenic predictions vs. 4 benign predictions) (PP3); the patient’s phenotype or family history is highly specific for a disease with a single genetic etiology (this variant was detected in two unrelated female patients diagnosed with bilateral breast cancer or breast and ovarian cancers, both with a strong family history of breast and ovarian cancer, and our patient showed a classical adenomatous polyposis phenotype with positive family history for the disease (PP4)); and well-established in vitro functional studies show no damaging effect on protein function [54] (BS3).

The proband was a woman of 58 years at the age of diagnosis who showed a classical FAP phenotype, with more than 100 adenomatous polyps at disease onset. The proband’s mother developed FAP at the age of 80 years, while the proband’s brother developed rectal cancer at the age of 60 years.

Patients n° 38 and 39 were carriers of only one heterozygous variant in the *NTHL1* gene, the *NTHL1*_ c.274C > T; p.Arg92Cys missense variant, which consists of a C to T transition at nucleotide 274, causing the substitution of the amino acid arginine at position 92 with a cysteine. This variant is reported in the ClinVar database and classified as conflicting interpretation of pathogenicity, with 8 submissions as VUS and 5 as likely benign [55,56,57,58,59,60]. In agreement with ACMG standards and guidelines for the interpretation of sequence variants [27], this variant is classified as a VUS. Indeed, there is 1 supporting line evidence of pathogenicity and 1 strong line of evidence of benignity: multiple lines of computational evidence support a deleterious effect on the gene or gene product (8 prediction pathogenic, uncertain, 3 benign, as reported by Varsome) (PP3); and the allele frequency is greater than expected for the disorder (BS1).

Patient n° 38 was a man of 55 years at the time of diagnosis. He developed about 15 intestinal adenomas at the age of 55 years and melanoma at the age of 53. He presented a congenital infrarenal aortic aneurysm, also reported for his father.

Patient n° 39 was a woman of 55 years at the age of diagnosis who did not develop gastrointestinal adenomas, while she showed a complex familial phenotypic manifestation. Further, she developed uterine cancer and was affected by ulcerative colitis, developing inflammatory polyps. Her sibling developed intestinal polyps, lipomas, and prostate calcifying lesions, while her daughter developed a thyroid cancer at the age of 25. Unfortunately, we could not screen the proband’s relatives.

Proband n° 40 was a carrier of the *AXIN2*_c.623C > T; p.Ala208Val variant, consisting of a C to T transition at nucleotide 623, causing the substitution of the amino acid alanine at position 208 with a valine, an amino acid with similar properties. This variant is reported in the ClinVar database and classified as conflicting interpretation of pathogenicity, with 3 submissions as VUS and 6 as likely benign/benign [60,61,62,63]. Although in silico analysis supports this missense variant as a tolerated alteration, not altering protein structure/function, it was observed in individuals with a personal or family history of colorectal and other cancers [61,62,63]. In agreement with ACMG standards and guidelines for the interpretation of sequence variants [27], this variant is classified as benign/likely benign. Indeed, there are 1 strong and 2 supporting lines of evidence of benignity: the allele frequency is greater than expected for the disorder (GnomAD exomes South Asian allele frequency = 0.000661 is greater than 0.000515) (BS1); multiple lines of computational evidence suggest no impact on the gene or gene product (BP4); and it is a missense variant in a gene for which primarily truncating variants are known to cause disease (2 out of 3 non-VUS missense variants in gene *AXIN2* are benign) (BP1).

The proband showed an attenuated phenotype of FAP, with more than 10 adenomas at disease onset at the age of 61 years and dominant inheritance of the disease within the family.

Patient n° 41 was a carrier of the *AXIN2*_ c.1685C > T; p.Pro562Leu variant, which consists of a C to T transition at nucleotide 1685, causing the substitution of the amino acid proline at position 562 with a leucine. This variant is reported in the ClinVar database and classified as conflicting interpretation of pathogenicity, with 3 submissions as VUS and 9 as likely benign/benign [64,65,66,67]. In silico analysis supports that this missense variant is a tolerated alteration, not altering protein structure/function, and it was observed in individuals affected by melanoma [64]. In agreement with ACMG standards and guidelines for the interpretation of sequence variants [27], this variant is classified as likely benign. Indeed, there are 1 strong and 2 supporting lines of evidence of benignity: the allele frequency is greater than expected for the disorder (GnomAD exomes European (non-Finnish) allele frequency = 0.000615 is greater than 0.000515) (BS1); multiple lines of computational evidence suggest no impact on the gene or gene product (BP4); and it is a missense variant in a gene for which primarily truncating variants are known to cause disease (2 out of 3 non-VUS missense variants in gene *AXIN2* are benign) (BP1).

The proband, a woman of 48 years at the age of diagnosis, did not show adenomas, but she developed breast and endometrial cancer and reported a positive family history of breast, rectum, colon, endometrium, thyroid cancer, and gastrointestinal polyposis (see Table 3).

Patient n° 42 was a carrier of the *POLD1*_c.269A > G; p.Gly90Arg variant, which consists of a A to G transition at nucleotide 269, causing the substitution of the amino acid glycine (which is a neutral and polar amino acid) at position 90 with an arginine (which is a basic and polar one). This variant is reported in the ClinVar database and classified as a VUS (Variation ID: 469286). In agreement with ACMG standards and guidelines for the interpretation of sequence variants [27], this variant is classified as a VUS. Indeed, there is 1 moderate line of evidence of pathogenicity and 1 supporting line of evidence of benignity: this variant shows extremely low frequency in the gnomAD population database (PM2); and computational prediction tools unanimously support a benign effect on the gene (BP4).

The proband was a man of 40 years at the age of diagnosis who showed a classical FAP phenotype, with more than 100 colorectal adenomatous polyps at disease onset and dominant inheritance of FAP.

Patient n° 43 was a carrier of the *POLE*_c.4106A > G; p.Asn1369Ser variant, which consists of a A to G transition at nucleotide 4106, causing the substitution of the amino acid asparagine at position 1369 with a serine. This variant is reported in the ClinVar database and classified as a VUS (Variation ID: rs746524982). Algorithms developed to predict the effect of missense changes on protein structure and function (SIFT, PolyPhen-2, Align-GVGD) all suggest that this variant is likely tolerated. Algorithms developed to predict the effect of sequence changes on RNA splicing suggest that this variant may create or strengthen a splice site. In agreement with ACMG standards and guidelines for the interpretation of sequence variants [27], this variant is classified as a VUS. Indeed, there are 1 moderate and 1 supporting lines of evidence of pathogenicity and 1 supporting line of evidence of benignity: this variant shows extremely low frequency in the gnomAD population database (PM2); multiple lines of computational evidence support a deleterious effect on the gene or gene product (PP3); and it is a missense variant in a gene for which primarily truncating variants are known to cause disease (BP1).

The proband was a woman of 54 years at the age of diagnosis, who developed an attenuated form of FAP, with 3 colon adenomatous polyps at disease onset, and she exhibited autosomal dominant mendelian inheritance.

Patient n° 44 was a carrier of the *AKT1* _c.1260 + 5G > A variant (rs754417090), which consists of a G to A transition at nucleotide +5 in intron 3, at the acceptor splicing site. This variant is reported in the ClinVar database and classified as a VUS (Variation ID: 649972). It does not directly change the encoded amino acid sequence of the AKT1 protein but affects a nucleotide within the consensus splice site. Algorithms developed to predict the effect of sequence changes on RNA splicing suggest that this variant may disrupt the consensus splice site. In agreement with ACMG standards and guidelines for the interpretation of sequence variants [27], this variant is classified as a VUS. Indeed, there are 1 moderate and 1 supporting lines of evidence of pathogenicity: this variant shows extremely low frequency in the gnomAD population database (0.0009%) (PM2); and multiple lines of computational evidence support a deleterious effect on the gene or gene product (PP3).

The proband was a woman of 56 years at the age of diagnosis who developed more than 10 colorectal adenomatous polyps and exhibited autosomal dominant inheritance for gastrointestinal cancers. Indeed, the proband’s mother died of gastric cancer, while a maternal aunt and maternal grand-aunt developed colon cancer.
