# Supplementary material for: Molecular Basis of Adenomatous Gastrointestinal Polyposis Syndromes: Role of Pathogenic and Benign Variants in Disease Onset
Source: Biomedicines. 2026 Feb 13;14(2):426. doi: 10.3390/biomedicines14020426 (PMC12938753; doi:10.3390/biomedicines14020426)

Figure S1. Total variants HeatMap. Heatmap showing variants identified in each sample. Red color: presence, blue color: absence of the specific variant. Variant positions are reported according to GRCh37.

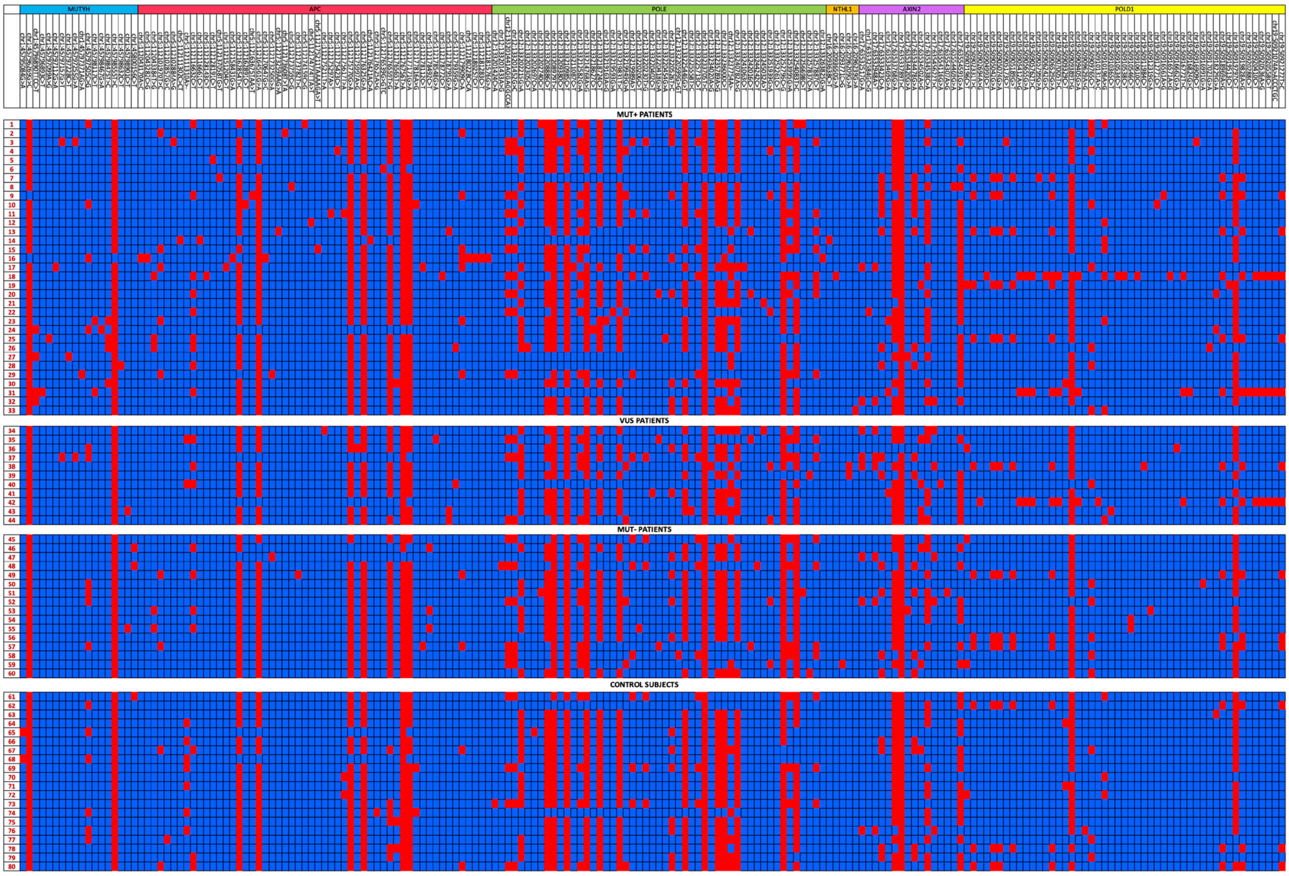

Supplement: Supplementary file 1 [file biomedicines-14-00426-s001.zip › supplementary materials/Figure S1.pdf]
