# Supplementary material for: Molecular Basis of Adenomatous Gastrointestinal Polyposis Syndromes: Role of Pathogenic and Benign Variants in Disease Onset
Source: Biomedicines. 2026 Feb 13;14(2):426. doi: 10.3390/biomedicines14020426 (PMC12938753; doi:10.3390/biomedicines14020426)
Supplement: Supplementary file 1 [file biomedicines-14-00426-s001.zip › supplementary materials/Table S1.pdf]

Table S1. Oligonucleotide designed for PCR (Polymerase chain reaction) and Sanger sequence of POLE, NTHL1 and AXIN2.

GENE	PRIMER	SEQUENCE
**POLE**	POLE_g.2FP	GCATTACAAATTAAGCACGGGG
**POLE**	POLE_g.2RP	AAGGACCACGCTATGACCAG
**POLE**	POLE_g.32FP	GCAGGTATAGACCTTGATCC
**POLE**	POLE_g.32RP	ATCCCCATAAGGTACTGAG
**NHTL1**	NTHL1_g.1FP	GGGAGTTGTAGTTCTGTGC
**NHTL1**	NTHL1_g.1RP	GGACCGCAATCTTTG
**NHTL1**	NTHL1_g.2FP	CCCCAAGTGTATTAGATGAT
**NHTL1**	NTHL1_g.2RP	GGTGCCAGCCAAAAG
**AXIN2**	AXIN2_g.1FPb	GTTGAAAAGCCTGTT
**AXIN2**	AXIN2_g.1RPb	CCAATAAGGAGTGTAAGGAC
**AXIN2**	AXIN2_g.5'UTRa	GAAATAAAAATAACC
**AXIN2**	AXIN2_g.1cRPa	GGGAATCCGGAGATG
**AXIN2**	AXIN2_g.1cRPb	CCAACCCATCTTCGT
**AXIN2**	AXIN2_g.8FP	TCATGTTTTGGCACTGACCC
**AXIN2**	AXIN2_g.8RP	TGAGACCCAGGCAGAAAGAG
